# Prediction factors and clinical significance of different types of hemorrhagic transformation after intravenous thrombolysis

**DOI:** 10.1186/s40001-023-01503-x

**Published:** 2023-11-11

**Authors:** Yanan Hao, Huan Zhou, Chengzhen Pan, Guomin Xie, Jin Hu, Bing Zhang, Shuxia Qian, Shenqiang Yan, the CASE II investigators

**Affiliations:** 1 Department of Neurology, The Second Affiliated Hospital of Jiaxing University, Jiaxing, China; 2 Department of Neurology, The Second Affiliated Hospital of Zhejiang University, School of Medicine, Hangzhou, China; 3 Jiaxing University Master Degree Cultivation Base, Zhejiang Chinese Medical University, Hangzhou, China; 4 Department of Neurology, Lee Hui-Lee East Hospital, Ningbo, China; 5 Department of Neurology, The First Affiliated Hospital of Jiaxing University, Jiaxing, China; 6 Department of Neurology, Huzhou Central Hospital, Huzhou, China

**Keywords:** Ischemic stroke, Thrombolytic Therapy, Cerebral Hemorrhage

## Abstract

**Background and purpose:**

Hemorrhagic transformation (HT) after intravenous thrombolysis (IVT) in acute ischemic stroke seriously affects the prognosis of patients. This study aimed to investigate the risk factors of different types of HT and their correlation with prognosis after IVT.

**Methods:**

Based on the CASE II registry, we included patients with acute ischemic stroke who received IVT within 4.5 h of onset. HT was further divided into hemorrhagic infarction (HI) and parenchymal hemorrhage (PH). Poor outcome was defined as a modified Rankin Scale (mRS) score of 3–6 at 3 months. Multivariate logistic regression analysis was used to determine the independent influencing factors of HT subtypes and clinical outcome.

**Results:**

Among 13108 included patients, 541 (4.1%) developed HI and 440 (3.4%) developed PH. In multivariate analysis, age (OR 1.038, 95% CI 1.028 to 1.049, *p* < 0.001), atrial fibrillation (OR 1.446, 95% CI 1.141 to 1.943, *p* = 0.002), baseline diastolic pressure (OR 1.012, 95% CI 1.004 to 1.020, *p* = 0.005), baseline NIHSS score (OR 1.060, 95% CI 1.049 to 1.071, *p* < 0.001) and onset to treatment time (OR 1.002, 95% CI 1.000 to 1.004, *p* = 0.020) independently predicted PH after IVT. In the patients with HT, PH (OR 3.611, 95% CI 2.540 to 5.134, *p* < 0.001) and remote hemorrhage (OR 1.579, 95% CI 1.115 to 2.235, *p* = 0.010) were independently related to poor outcome.

**Conclusions:**

Different types of HT after IVT had different risk factors and clinical significance. The occurrence of PH and remote hemorrhage independently increased the risk of poor outcome.

**Supplementary Information:**

The online version contains supplementary material available at 10.1186/s40001-023-01503-x.

## Introduction

At present, intravenous thrombolysis (IVT) with alteplase is the standard treatment for acute ischemic stroke [1]. Despite the transient symptom improvement of IVT, hemorrhagic transformation (HT) after thrombolysis is a serious complication, which increases the risk of poor prognosis [2, 3]. Geet al. showed that HT occurred in approximately 10–30%, which seriously affects the thrombolytic rate and clinical outcome [4]. A meta-analysis of 14 studies showed that age, atrial fibrillation, National Institutes of Health Stroke Scale (NIHSS) score before thrombolysis, previous stroke history, prior antiplatelet use, systolic or diastolic pressure before thrombolysis and blood glucose levels were risk factors affecting HT after IVT [5]. If a patient undergoes endovascular therapy, operation-related factors can also influence HT, such as the number of device passes [ref1, ref2]. Previously published studies of risk prediction in thrombolysis-related HT were designed for a less specific subtypes of hemorrhage transformation, that is, any type and size of hemorrhage. Nevertheless, each type of HT has different effect on prognosis. The European Cooperative Acute Stroke Study (ECASS)-II study divided HT into hemorrhagic infarction (HI) and parenchymal hematoma (PH) based on imaging CT findings [6]. PH-2 type has been confirmed by multiple studies to seriously affect the prognosis of patients, and its case fatality rate can reach 50% [7, 8].

The purpose of this study is to explore the possible risk factors predicting different subtypes of HT, especially for PH type that may cause distinct deterioration in neurological status and functional outcome, in a large multicenter stroke database.

## Methods

### Study subjects

The Computer-Based Online Database of Acute Stroke Patients for Stroke Management Quality Evaluation (CASE II) is a prospective multicenter registration study that aims to establish an online database of acute stroke patients for stroke management quality evaluation in China (NCT04487340). Based on CASE II registration, we included patients with acute ischemic stroke who received IVT within 4.5 h of stroke symptom onset from December 2016 to July 2021. We excluded patients who (1) were treated with endovascular therapy after IVT; (2) had no follow-up images. (3) experienced HT other than the types of HI and PH, such as remote hemorrhage, subarachnoid hemorrhage, or intraventricular hemorrhage; (4) lost to follow-up at 90 days.

### Clinical data

The following patient characteristics were recorded from the registry database, such as demographics, vascular risk factors, prior antithrombotic (antiplatelet and anticoagulant) drugs usage, onset to treatment time (OTT), baseline National Institutes of Health Stroke Scale (NIHSS) score, site and type of HT.

The head CT/MR imaging taken 24 h after IVT was reviewed and evaluated by radiologist and neurologists, with a consensus reached in case of discrepancies. Referring to the ECASS-II classification standard [6], HT was divided into HI and PH. HI is defined as petechial infarction without mass effects, while PH is defined as hemorrhage with mass effects [9, 10].

### Study outcomes

The primary outcome of this analysis was modified Rankin Scale (mRS) score at 90 days (good outcome defined as a score of 0–2 and poor outcome defined as a score of 3–6). Secondary outcomes were mortality within 7 days, and stroke recurrence at discharge and 90 days.

### Statistical analysis

Study participants with HT were dichotomized HI and PH. Patients without HT at the same time were used as the control group. Continuous variables were expressed as mean with standard deviation (SD), while median (interquartile range [IQR]), or No. (%) for ordinal variables. Independent samples *t* test or Mann–Whitney *U* test was used for the continuous variables, and Chi-square test or Fisher's exact test was used to compare the dichotomous variables between groups, while as appropriate. One-way ANOVA or Kruskal–Wallis test was used between multiple groups. Variables with a *p* value of < 0.1 in univariate analyses were included in the binary logistic regression model. All statistical analyses were carried out with SPSS 24.0 statistical software, and the difference was statistically significant with *p* < 0.05.

## Results

**Fig. 1:**
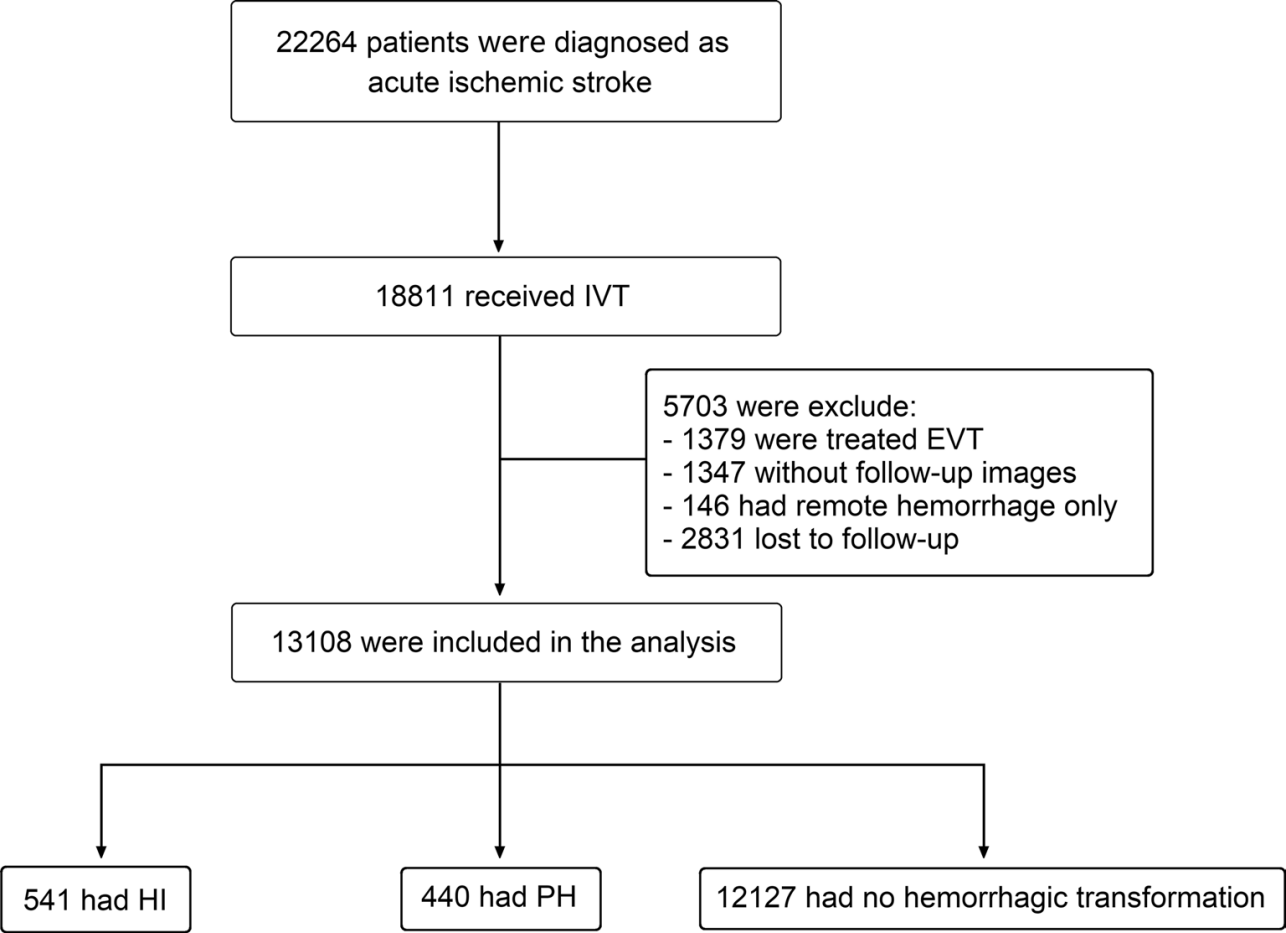
Flowchart of enrollment, treatment, and imaging profile. *HI* hemorrhagic infarction, *PH* parenchymal hemorrhage, *EVT* endovascular therapy, *IVT* intravenous thrombolysis

The flowchart of enrollment is shown in Fig. 1. From December 2016 to July 2021, a total of 18811 patients with acute ischemic stroke received IVT within 4.5 h from symptom onset in CASE II registration study. Among them, 1379 patients were treated with endovascular therapy, 1347 patients did not have follow-up images, 146 patients had remote hemorrhage only, and 2831 patients were lost to follow-up. The main reasons for missing follow-up images include early discharge due to excellent neurological recovery, patient refusal for further examinations due to personal reasons, and the inability to conduct comprehensive examinations due to severe deterioration of the patient's condition. Thus, 13108 patients were finally included in this analysis, among which, 981 (7.5%) had HT (4.1% for HI and 3.4% for PH). Of the included patients, the mean age was 69.5 ± 12.7 years and 8100 (61.8%) were male; the median baseline NIHSS was 5 (3–10) and median OTT was 149 (109–197) min. The median mRS score at 90 days is 3 in HI, but 5 in PH. 428 (3.3%) patients died within 7 days. 259 (2.0%) patients had stroke recurrence at discharge, and 193 (1.5%) at 90 days.

### Comparison of baseline data and prognosis stratified by the different types of HT

The baseline characteristics and functional outcomes stratified by HT type are shown in Table 1. Some baseline characteristics including age, sex, atrial fibrillation, smoking, use of antiplatelet agents and anticoagulant, baseline systolic pressure, baseline diastolic pressure, baseline NIHSS score, OTT and the incidence of combined remote hemorrhage had reached statistical differences (all *p* < 0.05) among patients with HI and PH, and the control group. The 7-day mortality, stroke recurrence rate at discharge, and 90 day mRS score were higher in patients with PH. The baseline characteristics and functional outcomes stratified by presence of HT are shown in Additional file 1: Table S1.

**Table 1:** Comparison of baseline characteristics and outcomes of patients with different types of HT

Variables	Control (12127)	HI (541)	PH (440)	P Value
Age (year)	70 (60–79)	76 (68–82)	78.5 (71–84)	**< 0.001**
Male, %	7532 (62.1)	314 (58.0)	254 (57.7)	**0.033**
Medical history				
Hypertension, %	7884 (65.0)	363 (67.1)	300 (68.2)	0.250
Diabetes mellitus, %	2037 (16.8)	92 (17.0)	65 (14.8)	0.529
Hypercholesterolemia, %	740 (6.1)	26 (4.8)	26 (5.9)	0.460
Atrial fibrillation, %	1793 (14.8)	178 (32.9)	153 (34.8)	** < 0.001**
Previous ischemic stroke, %	1572 (13.0)	70 (12.9)	69 (15.7)	0.252
Smoking, %	3031 (25.0)	111 (20.5)	84 (19.1)	**0.001**
Prior drugs use				
Prior antiplatelet use, %	1749 (14.4)	92 (17.0)	94 (24.1)	** < 0.001**
Prior anticoagulant use, %	169 (1.4)	15 (2.8)	11 (2.5)	**0.008**
Prior statin use, %	1154 (9.5)	43 (7.9)	54 (12.3)	0.068
Baseline systolic pressure (mmHg)	154 (140–168)	158 (140–172)	157 (143–170)	**0.001**
Baseline diastolic pressure (mmHg)	85 (76–94)	86 (77–96)	86 (78–95)	**0.006**
Baseline NIHSS score	5 (2–9)	11 (5–16)	12 (6–18)	** < 0.001**
OTT (min)	149 (108–196)	157 (115–207)	155 (110–206)	**0.009**
Remote hemorrhage	NA	163 (30.1)	249 (56.6)	** < 0.001**
Fibrinolytic drug				0.519
rt-PA, %	11920 (98.3)	534 (98.7)	435 (98.9)	
Urokinase, %	207 (1.7)	7 (1.3)	5 (1.1)	
7d mortality, %	271 (2.2)	42 (7.8)	115 (26.1)	** < 0.001**
Stroke recurred at discharge, %	222 (1.8)	11 (2.0)	26 (5.9)	** < 0.001**
Stroke recurred at 3 m, %	184 (1.5)	6 (1.1)	3 (0.7)	0.259
mRS score at 90d	1 (0–3)	3 (1–5)	5 (3–6)	** < 0.001**

*HI* hemorrhagic infarction, *PH* parenchymal hemorrhage, *OTT* onset to treatment time, *mRS* modified Rankin Scale. Bold text indicates a p-value < 0.05

### Logistic multivariate analysis of HT after IVT

The predictors with marginally significant difference (*p* < 0.1) in univariate analysis were estimated using binary logistic regression analysis, as shown in Tables 2 and 3. Older age, higher baseline diastolic pressure and baseline NIHSS score, and longer OTT were associated with a higher risk of HT. Older age, higher baseline diastolic pressure and baseline NIHSS score, longer OTT, and comorbid atrial fibrillation were associated with a higher risk of PH.

**Table 2:** Logistic multivariate analysis for HT after IVT (No HT [12127] vs HT [981])

Variables	OR	95% CI	*p* Value
Age	1.029	1.022–1.036	** < 0.001**
Male	1.104	0.950–1.284	0.197
Atrial fibrillation	1.643	1.390–1.943	** < 0.001**
Smoking	1.032	0.858–1.242	0.740
Prior antiplatelet use, %	1.261	1.014–1.567	**0.037**
Prior anticoagulant use, %	1.219	0.780–1.905	0.386
Prior statin use, %	0.747	0.566–0.986	**0.040**
Baseline systolic pressure	0.999	0.996–1.003	0.753
Baseline diastolic pressure	1.013	1.007–1.018	** < 0.001**
Baseline NIHSS score	1.055	1.047–1.063	** < 0.001**
OTT (min)	1.002	1.001–1.004	** < 0.001**

*OR* odds ratio, *CI* confidence interval, *OTT* onset to treatment time. Bold text indicates a p-value < 0.05

**Table 3:** Logistic multivariate analysis for PH after IVT(NO PH [12668] vs PH [440])

Variables	OR	95% CI	*p* Value
Age	1.038	1.028–1.049	** < 0.001**
Male	1.155	0.930–1.832	0.194
Atrial fibrillation	1.446	1.141–1.943	**0.002**
Smoking	1.054	0.803–1.383	0.705
Prior antiplatelet use, %	1.314	0.969–1.783	0.079
Prior anticoagulant use, %	1.136	0.594–2.174	0.700
Prior statin use, %	0.948	0.652–1.378	0.780
Baseline systolic pressure	1.000	0.994–1.005	0.855
Baseline diastolic pressure	1.012	1.004–1.020	**0.005**
Baseline NIHSS score	1.060	1.049–1.071	**< 0.001**
OTT (min)	1.002	1.000–1.004	**0.020**

*OR* odds ratio, *CI* confidence interval, *OTT* onset to treatment time. Bold text indicates a p-value < 0.05

### Factors influencing the clinical outcome of patients with HT after IVT

Table 4 shows the baseline characteristics of patients stratified by primary outcome. Multivariate logistic regression analysis resulted in 6 risk factors that independently associated with clinical outcome in the patients with HT. Patients with PH was more likely to have a poor outcome compared with those with HI (25.4% vs 55.2%, OR 3.611, 95% CI 2.540 to 5.134, *p* < 0.001). In addition, combination of remote hemorrhage also independently increased the risk of poor outcome (OR 1.579, 95% CI 1.115 to 2.235, *p* = 0.010) (Table 5).

**Table 4:** Comparison of baseline characteristics in patients with HT after IVT

Variables	mRS 0–2 (342)	mRS 3–6 (639)	*p* Value
Age (year)	73 (64–79)	80 (72–85)	** < 0.001**
Male, %	222 (64.9)	346 (54.1)	**0.001**
Medical history			
Hypertension, %	224 (65.5)	439 (68.7)	0.317
Diabetes mellitus, %	51 (14.9)	106 (16.6)	0.523
Hypercholesterolemia, %	12 (3.5)	40 (6.3)	0.073
Atrial fibrillation, %	83 (24.3)	248 (38.8)	** < 0.001**
Previous ischemic stroke, %	28 (8.2)	111 (17.4)	** < 0.001**
Smoking, %	80 (23.4)	115 (18.0)	**0.045**
Prior drugs use			
Prior antiplatelet use, %	53 (15.5)	133 (20.8)	**0.049**
Prior anticoagulant use, %	4 (1.2)	22 (3.4)	**0.037**
Prior statin use, %	30 (8.8)	67 (10.5)	0.433
Baseline systolic pressure (mmHg)	156 (140–169)	160 (143–172)	**0.026**
Baseline diastolic pressure (mmHg)	87 (77–96)	86 (78–95)	0.620
Baseline NIHSS score	6 (3–11)	14 (10–20)	** < 0.001**
OTT (min)	115 (110–209)	157 (113–206)	0.570
Remote hemorrhage	116 (33.9)	296 (46.3)	** < 0.001**
Fibrinolytic drug			0.677
rt-PA, %	339 (99.1)	630 (98.6)	
Urokinase, %	3 (0.9)	9 (1.4)	
Hemorrhage type			** < 0.001**
HI, %	255 (74.6)	286 (44.8)	
PH, %	87 (25.4)	353 (55.2)	

*mRS* modified Rankin Scale, *HI* hemorrhagic infarction, *PH* parenchymal hemorrhage, *OTT* onset to treatment time, *rt-PA* recombinant tissue plasminogen activator. Bold text indicates a p-value < 0.05

**Table 5:** Multivariate logistic regression analysis for clinical outcome in patients with HT after IVT (mRS 0–2 [342] vs mRS 3–6 [639])

Variables	OR	95% CI	p Value
Age	1.046	1.029–1.064	** < 0.001**
Male	0.829	0.577–1.190	0.309
Hypercholesterolemia	1.910	0.859–4.251	0.113
Atrial fibrillation	1.062	0.725–1.557	0.757
Previous ischemic stroke	2.168	1.258–3.738	**0.005**
Smoking	1.619	1.042–2.514	**0.032**
Antiplatelet agents	1.026	0.652–1.614	0.913
Anticoagulant	3.005	0.857–10.536	0.086
Baseline systolic pressure	1.004	0.996–1.012	0.279
Baseline NIHSS score	1.185	1.150–1.221	** < 0.001**
Remote hemorrhage	1.579	1.115–2.235	**0.010**
PH	3.611	2.540–5.134	** < 0.001**

*OR* odds ratio, *CI* confidence interval, *NIHSS* National Institutes of Health Stroke Scale. Bold text indicates a p-value < 0.05

## Discussion

In our study, 7.5% of thrombolysis patients developed HT, while PH accounted for 3.4%. Older age, higher baseline diastolic pressure and baseline NIHSS score, longer OTT, and comorbid atrial fibrillation independently predicted PH 24 h after IVT. Moreover, when we limit the target population to the patients with HT after IVT, the occurrence of PH and remote hemorrhage independently increased the risk of poor outcome.

HT can show a wide range from petechiae to hematoma. HI is considered as extravasation or capillary rupture bleeding after recanalization, while PH is bleeding caused by vascular rupture in ischemic area caused by reperfusion pressure and can cause space occupying effect or ventricular leakage [11–15]. There are many factors affecting HT after thrombolysis, but the prediction of risk factors for different types of HT has not been uniformly concluded. Previous studies found that male, age, previous stroke history, diabetes mellitus, baseline NIHSS score, OTT, rt-PA therapy, prior anticoagulant, infarct size, CT ischemic signs and atrial fibrillation were independent predictors for HT [16–18]. On the other hand, atrial fibrillation, hyperglycemia, baseline NIHSS score, antiplatelet agents and statins, degree of substantial low attenuation on baseline CT scan, history of congestive heart failure, older age and higher baseline systolic pressure were predictors of PH [16, 17, 19]. Our study found that age, prevalence of atrial fibrillation, baseline diastolic pressure, baseline NIHSS score and OTT were independently related to HT occurred 24 h after thrombolysis, so was for PH-type, which was consistent with previous researches [20, 21]. As the older the age is, the more vulnerable it is to be affected by the permeability of blood–brain barrier (BBB) during ischemia [22], the higher the burden of cerebral microbleeds [23, 24]. Hypertension damages vascular elasticity and excessive blood pressure can easily lead to hyperperfusion damage. High baseline NIHSS score indicates large cerebral infarction area which is prone to occur secondary severe cerebral edema, compressing the surrounding vessels and causing damage and inflammatory reaction of the involved vascular endothelial cells, increasing the permeability of the vessels, and more likely to cause post reperfusion hemorrhage when the vessels are re-passed [25]. The longer the OTT is, the longer the ischemia time is. Cerebral ischemia causes the explosion of oxygen radicals, the oxidation of phospholipids and fatty acids in the cell membrane, and the release of a large number of inflammatory factors, resulting in the destruction of the blood–brain barriered autoregulatory system of the cerebrovascular, which will easily lead to bleeding during reperfusion therapy.

Most studies have suggested that atrial fibrillation is a risk factor for spontaneous HT after ischemic stroke, and affects the outcome of patients [26, 27]. However, Lou et al. found that atrial fibrillation increased the risk of PH but not HI, and was not an independent risk factor for poor outcome in thrombolytic patients [28] Our study also found that atrial fibrillation could independently increase the risk of PH, which might be due to higher rate of anticoagulant therapy and larger infarct size. In addition, atrial fibrillation can lead to severe baseline cerebral hypoperfusion, coupled with the acute onset of cardiac embolism, so that collateral circulation cannot be fully established in a short time, aggravating the degree of hypoperfusion and local ischemia, which results in the vessels is damaged and vascular permeability is increased. When reperfusion therapy is performed, bleeding is more likely to occur [29]. Poor collateral circulation is significantly associated with HT, even in patients without large vessel occlusion. Of course, this relationship is more crucial in patients undergoing endovascular therapy [ref 1]. Our study found that prior antiplatelet therapy was independently related to HT, but not PH, which was supported by the findings of Zhong et al. [30].

Amarenco et al. found that statin therapy increased the risk of hemorrhagic stroke [31]. Bang et al. also found that statin therapy was associated with higher risk of symptomatic HT [32]. However, contrary to previous findings, the use of statins could reduce the risk of HT in our study. It is considered that statins may be related to inhibiting vascular endothelial inflammatory response, improving vascular microcirculation and vascular endothelial function [33].

The effect of HT on the outcome of acute ischemic stroke is related to its specific type. At present, it is recognized that patients with PH were more likely to have poor outcome [10, 17, 34] but not HI [35], and even the early occurrence of HI after thrombolysis may be a sign of vascular recanalization [36]. A systematic review and meta-analysis found that remote intracerebral hemorrhage (rICH) in acute ischemic stroke could lead to worse functional outcomes and higher mortality [37]. Our study also found that remote hemorrhage independently increased the risk of poor outcome. It is considered that small vessel disease markers (cerebral amyloid angiopathy [CAA], white matter hyperintensity [WMH], cerebral microbleed [CMB]) are associated with chronic endothelial dysfunction and the disruption of the BBB across the whole brain, affecting not only the WMHs area, but also the normal-appearing white matter and cortex [38, 39]. The pre-existing BBB disruption is exacerbated after intravenous thrombolysis with alteplase, and subsequently prompting rICH [40].

Our study has certain limitations. First, although we collected data prospectively through multicenter stroke registry, a retrospective design has potential risk of selective bias. Some patients did not undergo follow-up imaging due to changes in their condition (either significantly improved or deteriorated), which might affect the primary outcome of this study. Second, due to the non-mandatory nature of certain data submission to the CASE II registry, crucial factors such as baseline Alberta Stroke Program Early CT Score, admission blood glucose levels and international normalized ratio were not assessed. Moreover, potential risk factors linked to HT, such as biomarkers, were not incorporated into this analysis. Third, the CASE II registry encompasses hospitals of varying levels, where the influence of different operational tiers on HT might surpass that of traditional risk factors. Hence, this study concentrates on patients only undergoing IVT. However, excluding patients who received endovascular therapy clearly limits the potential clinical application.

## Conclusion

Our study shows that different types of HT after IVT had different risk factors and clinical significance. The occurrence of PH and remote hemorrhage independently increased the risk of poor outcome in those developed HT after IVT.

### Supplementary Information

**Additional file1: Table S1**. Comparison of baseline characteristics and outcomes of patients with or without HT.

## Data Availability

The data sets used and/or analysed during the current study are available from the corresponding author on reasonable request.

## Abbreviations

HT: Hemorrhagic transformation
IVT: Intravenous thrombolysis
HI: Hemorrhagic infarction
PH: Parenchymal hemorrhage
mRS: Modified Rankin Scale
NIHSS: National Institutes of Health Stroke Scale
ECASS: European Cooperative Acute Stroke Study
OTT: Onset to treatment time
rt-PA: Recombinant tissue plasminogen activator
rICH: Remote intracerebral hemorrhage
CAA: Cerebral Amyloid Angiopathy
WMH: White Matter Hyperintensity
CMB: Cerebral microbleed
BBB: Blood–Brain Barrier
SD: Standard Deviation
IQR: Interquartile range
OR: Odds Ratio
CI: Confidence Interval
