# Supplementary material for: Prediction factors and clinical significance of different types of hemorrhagic transformation after intravenous thrombolysis
Source: Eur J Med Res. 2023 Nov 11;28:509. doi: 10.1186/s40001-023-01503-x (PMC10638828; doi:10.1186/s40001-023-01503-x)
Supplement: Supplementary file 1 — Additional file1: Table S1. Comparison of baseline characteristics and outcomes of patients with or without HT. [file 40001_2023_1503_MOESM1_ESM.doc]

**Additional file 1: Table S1**. Comparison of baseline characteristics and outcomes of patients with or without HT

| Variables | Control (12127) | HT (981) | P Value |
| --- | --- | --- | --- |
| Age (year) | 70 (60-79) | 77(69-83) | **<0.001** |
| Male, % | 7532 (62.1) | 568（57.9） | **0.009** |
| Medical history |  | | |
| Hypertension, % | 7884 (65.0) | 663(67.6) | 0.104 |
| Diabetes mellitus, % | 2037 (16.8) | 157(16.0) | 0.522 |
| Hypercholesterolemia, % | 740 (6.1) | 52(5.3) | 0.311 |
| Atrial fibrillation, % | 1793 (14.8) | 331(33.7) | **<0.001** |
| Previous ischemic stroke, % | 1572 (13.0) | 139(14.2) | 0.281 |
| Smoking, % | 3031 (25.0) | 195（19.9） | **<0.001** |
| Prior drugs use |  | | |
| Prior antiplatelet use, % | 1749 (14.4) | 186(19.0) | **<0.001** |
| Prior anticoagulant use, % | 169 (1.4) | 26(2.7) | **0.002** |
| Prior statin use，% | 1154 (9.5) | 97(9.9) | 0.703 |
| Baseline systolic pressure (mmHg) | 154 (140-168) | 158(142-171) | **<0.001** |
| Baseline diastolic pressure (mmHg) | 85 (76-94) | 86(78-96) | **0.001** |
| Baseline NIHSS score | 5 (2-9) | 12(5-17) | **<0.001** |
| OTT (min) | 149 (108-196) | 156(111-207) | **0.002** |
| remote hemorrhage | NA | 412(42.0) | NA |
| Fibrinolytic drug |  |  | 0.256 |
| rt-PA, % | 11920 (98.3) | 969(98.8) |  |
| urokinase, % | 207 (1.7) | 12(1.2) |  |
| 7d mortality, % | 271 (2.2) | 157(16.0) | **<0.001** |
| Stroke recurred at discharge, % | 222 (1.8) | 37(3.8) | **<0.001** |
| Stroke recurred at 3m, % | 184 (1.5) | 9(0.9) | 0.134 |
| mRS score at 90d | 1 (0-3) | 4(1-6) | **<0.001** |

HI: hemorrhagic infarction; PH: parenchymal hemorrhage; OTT: onset to treatment time; mRS: modified Rankin Scale.
